# Mobility in Older Community-Dwelling Persons: A Narrative Review

**DOI:** 10.3389/fphys.2020.00881

**Published:** 2020-09-15

**Authors:** Ellen Freiberger, Cornel Christian Sieber, Robert Kob

**Affiliations:** Institute for Biomedicine of Aging, Friedrich-Alexander-Universität Erlangen-Nürnberg, Nürnberg, Germany

**Keywords:** mobility, gait, risk factors, older person, narrative review, community-dwelling

## Abstract

Due to the demographic changes and the increasing awareness of the role of physical function, mobility in older age is becoming an important topic. Mobility limitations have been reported as increasingly prevalent in older persons affecting about 35% of persons aged 70 and the majority of persons over 85 years. Mobility limitations have been associated with increased fall risk, hospitalization, a decreased quality of life, and even mortality. As concepts of mobility are multifactorial and complex, in this narrative review, definitions, physical factors, and their age-related changes associated with mobility will be presented. Also, areas of cognitive decline and their impact on mobility, as well as neuromuscular factors related to mobility will be addressed. Another section will relate psychological factors such as Fall-related psychological concerns and sedentary behavior to mobility. Assessment of mobility as well as effective exercise interventions are only shortly addressed. In the last part, gaps and future work on mobility in older persons are discussed.

## Introduction

Due to the demographic changes in the western world, healthy aging becomes an important issue on the individual as well as on a population level. Between 2015 and 2050, the proportion of the world’s population over 60 years will increase from 12 to 22% (WHO, 2015). The WHO defines healthy aging “as the process of developing and maintaining the functional ability that enables wellbeing in older age” (WHO, 2015). One important component of healthy aging is mobility. Self-reported mobility limitations are frequent among older persons but this prevalence varies due to different concepts and models. Nevertheless, mobility limitations are increasingly commonplace in older persons affecting approximately 35% of persons aged 70 and the majority of persons over 85 years (Cummings et al., 2014; Musich et al., 2018). Mobility limitations have been associated with an increased fall, disability, hospitalization, and mortality risk as well as decreased quality of life, and poor psycho-social health next to declining function (Shumway-Cook et al., 2005; Gill et al., 2006; Hardy et al., 2011; Lee et al., 2012; Rosso et al., 2013b). Hardy et al. (2011) demonstrated additional health care costs in older persons with mobility limitations. In older persons with mobility limitations, an additional $2773 (95% CI $1443–4102) in total health care expenditures, an additional $274 (95% CI $30–518) in out-of-pocket expenditures, and an additional 14 (95% CI 8–20) hospitalizations per 100 beneficiaries occurred (Hardy et al., 2011). These numbers demonstrate the need to understand the mechanism and risk factors for mobility limitations.

It is commonly understood that a low physical activity level has negative impact on health, and is responsible for many chronic diseases (Booth et al., 2012, 2017). A low activity level has been linked to sarcopenia (Freiberger et al., 2011; Marzetti et al., 2017a), and to mobility limitations (Gill et al., 2012; Brown and Flood, 2013). With regard to the fact that most people in the western world over 65 years do not meet the recommended physical activity level for healthy aging (Hallal et al., 2012), this area seems mandatory to include in preventing mobility limitations. Decreasing sedentary behavior or inactivity by maintaining mobility in older persons is, with regard to independence, mortality, and health in older persons, a priority on an individual as well as a population level. In 2009 the WHO stated that physical inactivity increased the risk for global mortality by 6% and was one of the four leading risk factors (WHO, 2018).

Ferrucci and others have even argued that mobility is a “hallmark of aging” and an important pillar for independent status (Ferrucci et al., 2016; Brabrand et al., 2018).

As mobility is such an important factor, two important issues occur: (1) early identification of mobility limitations in older persons and (2) the installation of effective interventions to modify or even reverse mobility limitations.

This narrative review will address on a broader base the mechanism and risk factors for mobility limitations, possible screening methods, and, briefly, on effective interventions.

## Definition of Mobility

Although at present, there is no gold standard definition of mobility in older persons, in most concepts and models mobility is understood as “one’s ability to move independently around their environment” (Mitchell et al., 2018). The theoretical framework by Webber et al. (2010) takes the different and complex determinants for mobility into account. Webber defines mobility “as the ability to move oneself (either independently or by using assistive devices or transportation) within environments that expand from one’s home to the neighborhood and to regions beyond” (Webber et al., 2010). The theoretical framework by Webber et al. (2010) includes multiple determinants of mobility, covering transportation/environmental aspects, cognitive, physical, financial, psychosocial, cultural, and gendered aspects. The included determinants demonstrate the need for holistic approaches in the area of mobility in older persons. As the interaction of these domains is dynamic over the aging process, it is mandatory that biology, medicine, and population science overcome boundaries and work together for a better understanding of the life course of mobility. Furthermore, the integration of older persons and their needs is vital for future mobility research.

**FIGURE 1:**
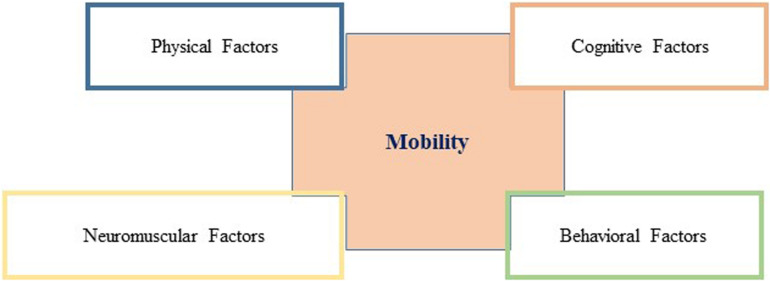
Age-related physiological changes with regard to mobility in older community-dwelling Persons.

As addressed above, mobility includes several domains ranging from physical, cognitive, and neuromuscular to psychological domains. On a physical level, gait, balance, and strength play an important role. In the neuromuscular domain, changes in the motor units are important, and in the cognitive domain, age-related changes are relevant as well as psychological factors such as fall-related psychological concern (FrPC). Figure 1 displays a conceptual model of these domains on the concept of mobility.

## Age-Related Physical Changes Associated With Mobility

### Age-Related Balance and Gait Changes

Postural control includes two domains: (a) static (balance) and (b) dynamic (gait) components. In the static condition the center of mass remains between the base of support whereas in gait the center of mass as well as the base of support shifts (Osoba et al., 2019).

### Balance Changes With Aging

With aging, postural control in the static condition, or balance, is influenced by the visual, sensory, and vestibular systems (Manchester et al., 1989; Choy et al., 2003). The decline of the sensory system occurs with increasing age and results in balance instability and gait limitations. Sensory feedback is necessary for balance control in the light of different environmental circumstances, e.g., different light situations such as sun or shadow, or traffic situations, e.g., sound recognition or localization (Cavazzana et al., 2018). Sensory feedback in static balance is necessary to reduce sway movement, e.g., in a situation where the room lights suddenly turn off, the upright position of the body needs feedback from other sensory systems.

A sensory decline occurs with aging especially for vision and hearing (Pinto et al., 2017). As declining hearing abilities as well as impaired vision have a negative impact on mobility, in addition it also has an impact on quality of life in older persons (Pinto et al., 2017; Liljas et al., 2020). The study by Pinto et al. (2017) in a US population demonstrated that no single sensory impairment had a negative effects on mobility –measured with the Timed-up and Go test (TUG) – but a global sensory index showed significant effects on mobility (Pinto et al., 2017).

### Gait Changes With Aging

In general, mobility limitations are characterized by temporal or spatial gait changes in numerous studies. In addition, gait has been used as a marker for physical function, as a predictor for falls, and even mortality (Studenski et al., 2011; Herssens et al., 2018). Gait is a highly complex process that is influenced by the central/peripheral nervous system, muscular skeletal changes, and by brain changes, e.g., the basal ganglia or the motor cortical regions (Montero-Odasso et al., 2012; Herssens et al., 2018; Clark et al., 2019).

One of the most used variables in aging research is gait speed (Studenski et al., 2011; Cruz-Jimenez, 2017). Gait speed can be measured in self-selected (often describes as “normal” or “usual”) gait and in fast gait speed to identify resources. Other gait variables are stride length or width and cadence (Herssens et al., 2018).

Early research by Winter et al. (1990) demonstrated a significant reduction in gait speed due to a shortened stride length in a study comparing self-selected gait in younger and healthy older persons. These early findings were later confirmed by Ko et al. (2010) adding the age-related changes in step width to the earlier findings. As usual, gait speed had been related to mortality by showing an 89% increased risk in mortality in older persons with the slowest gait speed (Liu et al., 2016). It has been suggested that gait is the “6th vital sign” in geriatrics (Fritz and Lusardi, 2009). A recent review by Herssens et al. (2018) confirmed the decrease in step length and step time, as well as an increase in stance with aging. In conclusion, evidence now exists that proves that spatio-temporal parameters in gait decline with age. In contrast, the question of any gender specific differences in gait changes with aging is less investigated.

Fast gait speed seems to decline at an earlier age then normal walking speed (Ko et al., 2010; Ferrucci et al., 2016; Callisaya et al., 2017). Fast gait speed is needed in daily life, e.g., in a timely pedestrian crossing. A walking speed of about >1.2 m/s is needed to cross safely during the green signal lights (Donoghue et al., 2016; Eggenberger et al., 2017). An Irish TILDA study demonstrated that one third of the population walked slower than the required 1.2 m/s (Donoghue et al., 2017) putting these older persons at stress when crossing a street. Eggenberger et al. (2017) presented similar findings in their study by showing that 30% of people in the age group 70–79 years and 73% of persons aged 80 years and older were not able to reach the 1.2 m/s threshold.

In the InCHIANTI study, Ferrucci et al. (2016) demonstrated that the above mentioned gait changes occurred at different stages over the life span. In their study cohort, the normal gait speed was stable in persons up to 65–70 years whereas the fast gait speed performance started to decline even after the age of 40–50 years.

Another important marker is the walking speed reserve, which is calculated by the difference between fast to normal gait speed (Callisaya et al., 2017). In a daily routine, catching the bus or keeping up with peers can require reserve capacity in gait speed. The walking speed reserve marker can become more important in the future. In the study by Callisaya et al. (2017) about 12.8% of the participants could not increase their gait speed to the level for a safe road crossing speed.

## Age-Related Neuromuscular Changes Associated With Mobility

On a muscular level, a change in muscle fiber sizes occurs with aging (Tieland et al., 2018). It is commonly understood that the type II fibers (fast twitch) are especially affected – they are responsible for generating the power in chair-rise performance (Tieland et al., 2018). Next to the reduction in muscle fiber size, other age-related changes are coming into the focus, e.g., the role of motor units (MU).

Motor units are responsible for the organization of neural control in any muscle. The MUs are composed of a single alpha motor neuron and the connected muscle fibers (Malafarina et al., 2012; Drey et al., 2014; Piasecki et al., 2016; Walker et al., 2018). The number of MUs can be estimated with the motor unit number index (MUNIX; Drey et al., 2014). At present, in the neuro-centric approach the loss of MUs is responsible for one pathophysiologic pathway of sarcopenia (Drey et al., 2014). In addition, there is an increase in the size of the surviving MUs (meaning an increased number of innervated muscle fibers per alpha motor neuron) reported to compensate for the MUs loss (Drey et al., 2014; Piasecki et al., 2016; Tieland et al., 2018). The compensation and remodeling process can lead to the re-innervation by axonal sprouting from other motor neurons (Tieland et al., 2018). Next to this process, a greater variability in MU discharge is reported (Tieland et al., 2018; Walker et al., 2018). A variety of firing rates, in muscles with reduced MU (up to 30–40%) are reported during maximal isometric contractions (Tieland et al., 2018).

Clark (2019) demonstrated that neural activation of skeletal muscle is a key component for muscle weakness. Other processes of interest are impaired voluntary muscle activation and/or increased antagonist activation (Russ et al., 2012; Clark, 2019).

In conclusion, morphological and physiological changes to MU due to aging is followed by alterations in the discharge properties of the MU (Russ et al., 2012; Clark, 2019).

### Age-Related Changes in Muscle Mass, Strength, and Power

Muscle performance declines with age (Doherty, 2001; Goodpaster et al., 2006; Brady et al., 2014; Ferrucci et al., 2014; Bell et al., 2016). Muscle performance is linked to muscle mass, muscle strength, and muscle power. Muscle strength is the ability to generate maximal muscle force whereas muscle power refers to the product of force and velocity of the muscle contraction (Reid and Fielding, 2012).

Muscle mass and strength have their peak on average in the third decade of life and slowly decline afterwards (Goodpaster et al., 2006; Ferrucci et al., 2016). Changes in muscle mass occur due to fat infiltration and loss of muscle fibers (Visser et al., 2005). Interestingly, evidence is accumulating that the loss of muscle mass and muscle strength deviate with advanced age (Goodpaster et al., 2006; Chen et al., 2013; Charlier et al., 2015). Muscle strength declines faster compared to the loss of muscle mass with a reduction of about 3 vs. 1% in older age (Bell et al., 2016). The loss of muscle mass can reach about 40% in persons older than 80 years but this decline can be modified by gender and lifestyle behavior. Furthermore, the national US sample by Chen et al. (2013) revealed that women have lower muscle mass and lower strength compared to their male counterparts.

Sarcopenia has formerly been recognized solely as the loss of muscle mass (Rosenberg, 1997). Due to the evidence that the loss of muscle mass is not congruent to the loss of muscle strength, the new definition of sarcopenia includes muscle mass and muscle quality with strength and gait parameters (Cruz-Jentoft et al., 2018). Based on the understanding that both muscle mass as well as strength have an impact on physical function, in the present sarcopenia definition, gait speed is included as a marker of physical performance (Cruz-Jentoft et al., 2018). Research has shown that sarcopenia is related to impairments in physical function, e.g., mobility limitations, and negative health outcomes as well as hospitalizations or falls (Cawthon et al., 2009; Brady et al., 2014; Cruz-Jentoft et al., 2018; Tieland et al., 2018; Cawthon et al., 2019).

The impact of muscle power on mobility in older age has been investigated with chair-rise or stair-climbing performance (Bell et al., 2016). With regard to strength decline, it seems that muscle power deteriorates on a faster slope. Reid et al. (2014) demonstrated in a longitudinal study that muscle power in their study population declined by about 3% per year. The rate of decline in muscle power seems to be 10% greater than the loss of muscle strength (Brady et al., 2014). Through research regarding muscle power and its impact on mobility limitation, a picture emerged that suggested muscle power (Reid and Fielding, 2012) has a higher impact on mobility than muscle strength (Bean et al., 2003, 2010; Trombetti et al., 2016). Research demonstrated that low leg power leads to a 2 to 3-fold higher risk of mobility limitation (Bean et al., 2003). Bean et al. (2003) revealed in their study that leg power was more related to reduced gait speed and chair-rise times then leg strength.

In conclusion, the role of muscle mass alone in mobility is less important than the role of muscle strength. Muscle strength only partly contributes to mobility but it especially contributes if strength is reduced in the lower extremity and when falling below a “threshold” it contributes to mobility limitations (Bean et al., 2003; Ferrucci et al., 2016). The role of muscle power is being looked at more in the present research. As it seems, muscle power has an even higher impact on mobility limitations than muscle strength (Reid and Fielding, 2012) and needs to be looked into closely when mobility in older age is investigated.

## Age-Related Changes in Cognition Associated to Mobility

In normal cognitive aging, the concepts of fluid and crystalized abilities describe different patterns of decline. Crystallized abilities include vocabulary and general knowledge, which the older person has gathered over their lifespan. With aging, the crystallized abilities remain stable for a long time (Harada et al., 2013).

In contrast, the fluid abilities including information processing speed, reasoning, and problem solving, declines with advancing age (Harada et al., 2013). The components of fluid abilities are especially important for mobility and will be described.

Processing speed starts to decline in the third decade of life, and continues with a linear decline over a life span (Harada et al., 2013; Salthouse, 2019). Processing speed is an important element for mobility, as the information from sensory input needs to be processed before the motor control system can adequately start.

In contrast to processing speed, attention and memory abilities show an accentuated decline with advancing age (Salthouse, 2019). The role of attention is especially important regarding mobility, as it is one important factor in gait. In combination, low attention followed by slow processing speeds might cause a hazard situation for balance or gait control.

Another important cognitive ability for balance and gait is executive function (EF). Executive functioning refers to capacities allowing an older person to successfully engage in independent, appropriate, and goal oriented behavior (Harada et al., 2013). Included in the EF concept are problem-solving abilities, and planning and organizing actions, which are cognitive elementary abilities with regard to mobility.

### Age-Related Relationship Between Gait and Cognition

In the last two decades, research has demonstrated that gait is no longer an “automatically controlled” but a cognitive influenced process. The first to demonstrate the “stops walking while talking” paradigm in relationship to falls was Lundin-Ollson in 1997. The “dual-task costs” arise from the additional cognitive task during gait performance (Lundin-Ollson et al., 1997; Verghese et al., 2013).

Emerging evidence shows that a decline in gait speed can predict cognitive decline by more than a decade (Verghese et al., 2013; Kikkert et al., 2016; Dumurgier et al., 2017; Montero-Odasso et al., 2018). The brain areas for gait control involve regions responsible for attentional, executive, and visuospatial functions as well as the cerebellum, basal ganglia, and motor cortex (Verghese et al., 2013; Holtzer et al., 2014; Kikkert et al., 2016; Demnitz et al., 2017; Wilson et al., 2019). Evidence is accumulating that proves there is an overlap between brain areas related to cognitive and gait decline (Verghese et al., 2013; Kikkert et al., 2016; Wilson et al., 2019). One of the important questions at present, originates from the complexity of cognition. The most investigated areas of cognitive function are EF (being responsible for planning and performing the movement), memory, and processing speed. All these cognitive areas have been related to gait and mobility (Rosso et al., 2013a,b). In a recent study, De Cock et al. (2019) demonstrated the relationship of spatiotemporal gait parameters with stages of cognitive impairments. They showed that the type of the additional cognitive task is essential to determine future dementia subtypes (De Cock et al., 2019).

In conclusion, gait and different gait variables are commonly used for quantifying mobility limitations. In addition, evidence of the predictive value of gait parameters with regard to cognitive decline is established (Kikkert et al., 2016).

## Risk Factors for Mobility Limitation

### Mobility and Chronic Diseases

Several studies have demonstrated that chronic diseases are a risk factor for mobility limitations (Welmer et al., 2013; Kujala et al., 2019). In the Twin study by Kujala et al. (2019) about 23.2% of their participants reported mobility restriction by a disease. Most commonly were musculoskeletal (60.2%), followed by cardiovascular (18.8%), and neurological disease (7.7%). Welmer et al. (2013) demonstrated in the Swedish National Study on Aging and Care that cardiovascular disease was followed by an increased odds ratio for mobility limitations. In their study mobility limitation was defined by a walking speed of 0.8 m/s.

As explained under the cognition section, neurological diseases such as dementia or mild cognitive impairments are related to mobility limitations (Demnitz et al., 2017, 2018).

### Physical and Cognitive Risk Factors for Mobility Limitation

Factors having an impact on muscle performance (muscle mass, strength, and power) are complex and multifactorial. Numerous research papers have described risk factors for sarcopenia (the loss of strength, muscle mass, and muscle performance) including a decreased anabolism pathway with sedentary lifestyle, bed rest, malnutrition, anorexia, age-related hormonal changes, and aging (Marzetti et al., 2017b). An increased catabolism pathway fostered by disease, injury, inflammation, oxidative stress, mitochondrial dysfunction, and an increase in myostatin also adds to the risk of sarcopenia (Narici and Maffulli, 2010; Morley et al., 2014; Marzetti et al., 2017b).

As described under age-related cognitive decline several cognitive capacities are also associated with mobility. The study by Pedersen et al. (2014) demonstrated higher mobility limitations in older persons with mild cognitive impairments. This was supported by Demnitz et al. (2018) who demonstrated an increasing relationship between cognition and mobility with advancing age. Furthermore, the INCHIANTI study (Ferrucci et al., 2016), showed that mobility limitations became evident even in the middle-aged group in challenging walking conditions supporting the influence of cognition on walking abilities even at an earlier age.

### Psychological Risk Factors for Mobility Limitation

It is commonly understood that next to physiological risk factors, lifestyle and psychological risk factors are coming into the picture of mobility in older persons (Gill et al., 2012; Brown and Flood, 2013).

One risk factor for mobility limitations least investigated is FrPC. Fall-related psychological concern is at present being used as an umbrella term including fear of falling, self-efficacy and balance related concern, and outcome expectance (Hughes et al., 2015; Payette et al., 2016; Pauelsen et al., 2018). Self-efficacy or balance related concerns address the individual thoughts of being able to cope with a situation, e.g., “I will not fall walking down the stairs,” whereas a fear of falling is a lasting concern that leads an older person to avoid activity irrespective of the capacities of the person (Delbaere et al., 2010; Hughes et al., 2015). Fall-related psychological concern has been investigated for decades with regard to falls but less in regard to mobility limitations. Fall-related psychological concern is present in about 55% of older persons, and more prevalent in women then in men (Pauelsen et al., 2018). FrPC has been strongly related to activity avoidance or declined physical activity, and social withdrawal thus contributing through an additional channel to mobility limitation (Hughes et al., 2015; Pauelsen et al., 2018). Older persons with FrPC reduce their activities to avoid falling or due to social embarrassment, e.g., asking for help to cope with environmental barriers. The association between FrPC and mobility has been only been investigated in the last few years (Auais et al., 2016, 2017; Litwin et al., 2018) demonstrating the strong association between FrPC and mobility decline. A barrier of investigating FrPC and mobility is the inconsistent use of concepts and terms. Confusion has been created by the interchangeable use of terms, e.g., fear of falling vs. self-efficacy (Hughes et al., 2015).

Another complex field of risk factors on mobility are, of course, environmental barriers (Levasseur et al., 2015; Cerin et al., 2017; Hinrichs et al., 2019). Reviews confirm the positive and negative impacts of a built environment, or the perceived environment by older persons on mobility.

**FIGURE 2:**
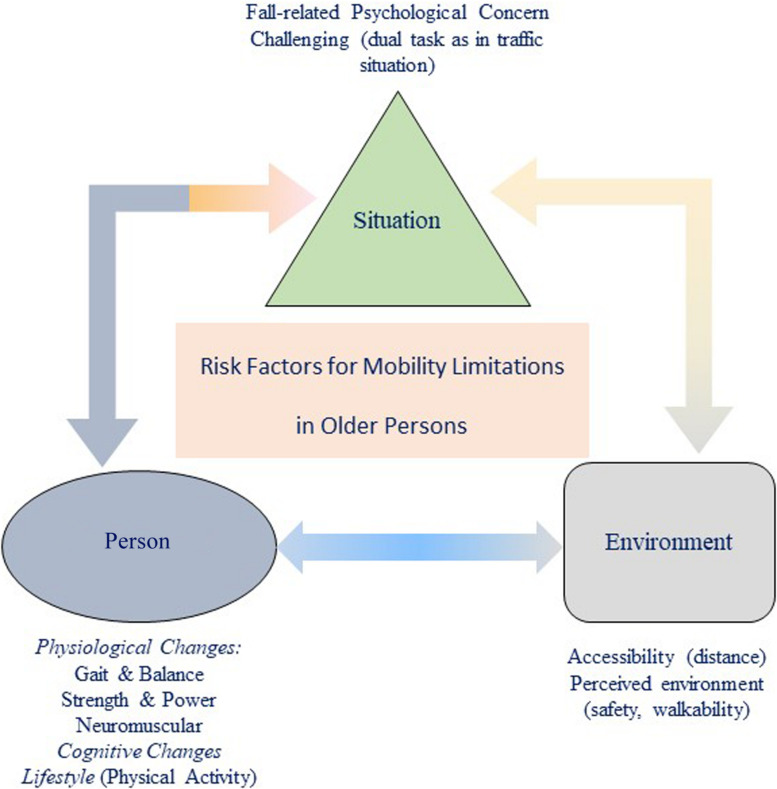
Interaction of risk factors for mobility limitation in older community-dwelling persons.

In conclusion, next to physiological risk factors, life style as physical activity, environment, and psychological factors such as FrPC pose an additional risk on mobility in older persons. The complexity of risk factors and mobility in older persons is outlined in Figure 2.

### Sedentary Behavior as a Risk Factor for Mobility Limitation

Research has demonstrated that older persons are more prone to sedentary behavior (Harvey et al., 2015; Loyen et al., 2017). Sedentary behavior is defined by the Sedentary Behavioral Research Network (SBRN) as “any waking activity characterized by an energy expenditure ≤ 1.5 metabolic equivalents while being in a sitting, reclining, or lying posture” (Tremblay et al., 2017). A recent systematic review (Scher et al., 2019) demonstrated that higher sedentary levels were related to mobility limitation. An interesting aspect in this area is that breaks or shorter periods of sedentary behavior have less negative impact on mobility limitations (Scher et al., 2019).

## Assessment of Mobility

Mobility is an important aspect of healthy aging and researchers as well as clinicians in a daily routine need effective and reliable assessment tools. The complex construct of mobility as well as the different courses to assess mobility in older persons makes different approaches in the assessment mandatory. Mobility measures are used for different reasons: (a) to screen for early mobility limitations at one time point or (b) to obtain changes in an individual’s mobility, e.g., after an intervention. Next to the purpose of assessment, available time and location also play an important role in the decision of assessment. The assessment of mobility in community-dwelling older persons range from self-reported mobility questionnaires (Brown and Flood, 2013; Taylor et al., 2019) or performance based measures to GPS obtained data (Fillekes et al., 2019). A new review of possible assessments for mobility has recently been performed by Soubra et al. (2019) including performance measures such as the well-known TUG, the Short Physical performance Test (SPPB), or the different walk tests (6-min, 2-min, or 400 m tests). Self-reported mobility ranges from simple questionnaires to life-space mobility (Brown and Flood, 2013; Taylor et al., 2019). Brown and Flood (2013) suggest stepwise questions by asking for difficulties in climbing up 10 stairs and walking - mile. If no difficulties are reported, a further question about modifications on climbing up 10 stairs (e.g., using the handrails) or in walking - mile (e.g., using an assistant device) should be provided.

As strength, balance, and gait are important components for mobility the SPPB is an excellent tool with good psychometric properties (Freiberger et al., 2012), and is often used in research as well as in the clinic to identify mobility limitations or functional decline (Gill et al., 2012; Pahor et al., 2014; Zunzunegui et al., 2015; Cruz-Jentoft et al., 2018).

Gait can be measured by a stopwatch and different lengths (ranging from 4 to 10 m to the 400 m walk) or with extensive technology. To show subclinical gait decline, the dual task paradigm is recommended (Beauchet et al., 2017). Evidence is accumulating that additional cognitive tasks, or tasks of increasing complexity, e.g., naming animals or reciting every second letter while walking/crossing over obstacles, is slowing down gait speed (Verghese et al., 2012; Kikkert et al., 2016; Muir et al., 2019). In many studies gait is obtained with modern technology based on sensors for spatial and temporal parameters (Oh-Park et al., 2010; Beauchet et al., 2017).

Fall-related psychological concerns are self-reported or obtained by questionnaires. One of the most used tools is the FES-I which was translated into many different languages (Yardley et al., 2005). Other questionnaires in this area are the SAFFEE and the ABC scale (Bladh et al., 2013).

## Exercise Intervention on Mobility

One of the most effective interventions in counteracting mobility limitation is exercise. Taking into account the physiological risk factors, it seems evident that an exercise intervention is based on strength, gait, and balance. The most effective interventions have addressed the muscle pathway by strength training exercises or in combination with balance and gait exercises (Martone et al., 2015; Marzetti et al., 2017a). One multidisciplinary, randomized, and controlled study contributing to the existing evidence of effective multicomponent exercise intervention on mobility prevention is the Lifestyle Interventions and Independence for Elders (LIFE) study by Pahor et al. (2014). The study addressed vulnerable and physically limited persons aged 70 years and older. The multicomponent exercises included walking, and strength and balance training. In the LIFE study, incidents of mobility disability – defined by the 400 m walk – were investigated demonstrating a significant positive effect in the prevention of mobility disability in the exercise group compared to the health education group. The intervention group experienced mobility disability at 30.1% in contrast to 35.9% in the health education group. Persistent mobility disability occurred in only 14.7% of the intervention versus 19.8% in the health education group (Pahor et al., 2014).

The LIFE study has been copiedby the European SPRINTT Study (*S*arcopenia & *P*hysical F*R*ailty *IN* older people: multi-componen*T T*reatment strategies) which is currently ongoing. SPRINTT uses the same methods and intervention program, so that data on mobility will be easily comparable at a later date (Landi et al., 2017; Marzetti et al., 2018).

In both studies, exercise intervention was individualized and tailored with regard to intensity including supervised and unsupervised sessions. The intervention was provided for between 24 and 36 months (Pahor et al., 2014; Marzetti et al., 2018).

One of the most disseminated and effective exercise programs is the OTAGO program developed by Campbell & Robertson in New Zealand. The first intervention was a home-based strength and balance exercise accompanied by a walking activity in women aged 80 years and older. Several other interventions with the same components (balance and strength exercises) replicated the positive effects on physical function, reduced fall rate, and other health outcomes (Campbell et al., 1999; Day, 2011). Later research demonstrated the effectiveness of the OTAGO program delivered as a group exercise (Kyrdalen et al., 2013). Although fall prevention was the first outcome of the OTAGO program, on a secondary level it also had positive effects on other health outcomes (Shubert et al., 2017). Overall the OTAGO program reduced falls by 32% and even reduced mortality over 12 months with a risk ratio of 0.48 (95% confidence interval 0.25–0.80) (Thomas et al., 2010).

A common component of the above-mentioned exercise programs are the structured format with increasing intensity and standardized repetitive exercises and the supervision and social feedback by experienced trainers. However, long-term adherence without this tight monitoring is questionable. Therefore, new concepts are developed to integrate exercise into daily routine. One approach would use a daily walking routine, e.g., walking to the store (Weber et al., 2018) whereas another approach would integrate functional exercises to help improve balance and strength in the daily routine (Clemson et al., 2012; Boulton et al., 2019). Integrating training exercises into a daily routine seems to have several advantages: requires no additional time to perform the exercise, includes a relationship to the daily routine (balance exercises, e.g., semi-tandem during cooking or washing), and improvements are linked to the daily routine thus enhancing motivation and compliance (Weber et al., 2018).

Newer concepts investigated the integration of video gaming to improve physical function but do not address mobility as a primary outcome.

## Discussion

Functional limitation increases with age and, due to demographic changes, early identification of older persons at risk is becoming mandatory (Tieland et al., 2018). Mobility is a major pillar of function, and mobility limitations in older community-dwelling persons are highly prevalent and followed by negative health events such as hospitalization or falls or even a higher mortality risk (Gill et al., 2006; Studenski et al., 2011; Brown and Flood, 2013; Musich et al., 2018). Research on mobility over the lifespan is rare and probably not feasible given the heterogeneity of mobility decline on an individual level and the complexity of factors involved (Ferrucci et al., 2016). Nevertheless, screening for early onset of mobility decline in older persons with regard to healthy aging and quality of life is without any alternative. Several important components of mobility in older persons were addressed in this narrative review. Several gaps remain which should be addressed in the future to move the research on mobility in older persons forward.

### Gaps to Be Addressed: Definitions and Concepts

To push the mobility research in older persons further, firstly an agreement of a concept as well as definition, and standardized assessment tools are needed (Rosso et al., 2013a; Varma et al., 2016; Beauchet et al., 2017; Cornman et al., 2019; Donoghue et al., 2019). With regard to definitions, these should not only be provided by researchers and clinicians but also by the older persons themselves. Intervention studies found that integrated adapted mobility strategies, e.g., taking longer to walk to the shop, might not be recognized by an older person. This activity is not –in an older person’s perception- related to mobility but to shopping, and therefore does not provide any understanding or motivation for improving mobility. Raising awareness of the components of mobility limitations to older persons by installing definitions and concepts needs to be addressed in the future.

Mobility in older age should not only be directed by disease-specific approaches but take into account the intrinsic capacity approach by the WHO (2015) including function and functional reserve (Cesari et al., 2018). Especially, the evidence that under challenging conditions mobility limitations occur even in apparently healthy older persons underlines the importance of the assessment of the functional reserve capacity.

From a scientific perspective, additional barriers arise from the use of different terms and mobility outcomes (mobility vs. walking or life-space mobility vs. functional mobility), posing a challenge when comparing different results and data.

### Gaps to Be Addressed: Physical Function and Mobility

Slow gait speed, and decline in muscle strength and power have all negative effects on mobility. Numerous studies have shown that having a higher level of physical function prevents mobility limitations (Guralnik et al., 2000). Exercise intervention, e.g., strength training, has demonstrated positive effects on functional mobility (Papa et al., 2017).

Nevertheless, a recent statement showed that the focus on musculoskeletal mechanisms and processes might not be the equivalent approach to counteract mobility limitations (Clark et al., 2019). The role of muscle mass related to mobility is much less than earlier anticipated. There is accumulating evidence that neuronal changes are more important in the process of mobility decline than biomechanical age-related changes.

In contrast to the existing literature on physical function and mobility, little evidence exists as to whether mobility decline occurs in a linear, dynamic, or even a mixed trajectory (Gill et al., 2006; Ahmed et al., 2019). Gill et al. (2006) have demonstrated that transitions between different mobility levels are very dynamic and it can be dangerous to estimate mobility limitations at only one point in time. The individual might revert back to their previous level of mobility limitation for different reasons (e.g., normal recovery after an injurious fall, or hip replacement, shortly after hospitalization) or develop an even more severe mobility limitation level.

### Gaps to Be Addressed: Cognition Brain and Mobility

One important topic that needs further investigation is the interaction between structural and functional brain changes and mobility in older persons. An initiative utilizing three workshops (Rosso et al., 2013a; Sorond et al., 2015; Varma et al., 2016) addressed this topic. New technological equipment such as magnetic resonance imaging (MRI) or functional MRI (fMRI) are being used. Functional near-infrared spectroscopy (fNIR) helped investigate which brain areas are involved in mobility and found evidence that the basal ganglia, cerebellum, and the frontal and parietal cortex are involved (Holtzer et al., 2014). Nevertheless, the specific brain regions, and neuronal networks involved in mobility need further clarification. The variability on the functional level in the older population – ranging from healthy/fit to disability/immobile – adds another important aspect to this topic. Up to now, no investigation on the impact of different functional levels in the research area of brain structures and mobility has been conducted. Further questions remain on the specific relationship between single gait parameters and cognitive variables. At present, research is investigating the involved brain regions related to mobility (Rosso et al., 2013a; Holtzer et al., 2014; Demnitz et al., 2017; Van Ooteghem et al., 2019).

### Gaps to Be Addressed: Neuromuscular Factors Related to Mobility

As Clark et al. (2019) concluded their editorial (2019) with the question “are we barking up the wrong tree?” future research has to take into account the area of neuromuscular changes. This will be an upcoming field for new evidence in the near future, as it seems to explain functional changes more than muscle mass. Furthermore, the question of past exercise experience over a lifetime – e.g., playing tennis – on intramuscular coordination with aging has yet to be integrated.

### Gaps to Be Addressed: Psychological and Behavioral Aspects

Another less investigated topic is the psychological aspects of mobility. In older persons, perceived mobility is related to personal experience and to psychological components such as FrPC (Goins et al., 2015). In addition, FrPC should be included in mobility research as it does not only moderate the physical activity level or risk of falling, but it also might act on a pathophysiology level by increasing inflammation and thus acting on the muscular pathway.

Furthermore, the motivation to change behavior in mobility is linked to intrapersonal, interpersonal, and environmental factors (Yardley et al., 2007; Goins et al., 2015). Changes in mobility strategies can be perceived as a support, e.g., using a wheeled walker to be able to still go out for shopping, or as a barrier, e.g., using a wheeled walker is perceived as an embarrassment, demonstrating the two-fold nature of the assistant device. Other elements to be recognized are self-efficacy, attitudes, and fear of falling (Hughes et al., 2015) as low self-efficacy – having low confidence in one’s abilities – will pose a barrier for the uptake of an appropriate intervention.

Another important aspect with regard to health and physical activity behavior is a positive self-perception on aging by older persons (Wurm et al., 2010; Wolff et al., 2014). Integrating such a “positive self-perception” component, e.g., in exercise intervention programs, might be an additional asset to motivate older persons into being more mobile (Beyer et al., 2019).

### Gaps to Be Addressed: Exercise Intervention and Mobility

Although there are exercise interventions being carried out on mobility, or are just finishing as with the EU SPRINTT project, several questions remain. In the LIFE study (Pahor et al., 2014), intervention was most effective in participants having an SPPB score of between 3 and 7 but was less effective in participants with an SPPB score of 8–9. This opens the question: which older person will profit most from an exercise intervention to maintain mobility? This question might depend on the time period of a follow up. In longitudinal observational studies such as one by Ferrucci et al. (2016) it becomes obvious that, depending on the demanding level of mobility, decline occurs at an earlier age but can be compensated. Furthermore, as there seems to be a gender aspect, future research should be conducted to investigate effects in both genders separately.

Another aspect regarding exercise intervention is the question of “how and if” exercise intervention has an impact on the intramuscular coordination by addressing neuromuscular age-related changes.

### Gaps to Be Addressed: Assessments

Most physical assessments, e.g., gait or walking performance, are obtained in the lab or under clinical conditions. Research suggests that this might not reflect the real physical capacity. Hillel et al. (2019) demonstrated that in comparison gait parameters under “real-world” conditions were much worse than in the lab-measured gait parameters. The development of new technology to obtain mobility data under “real-world” conditions such as the newly started EU Project “Mobilise-D” (connecting digital mobility assessment to clinical outcomes for regulatory and clinical endorsement) will in the future close this gap. The underlying thesis of Mobilise-D is that loss of mobility (slower walking, fewer steps per day, or more time sitting) predicts adverse medical outcomes regardless of underlying disease such as chronic obstructive pulmonary disease, Parkinson’s disease, multiple sclerosis, hip fracture recovery, and heart failure. The frame of Mobilise-D is that loss of mobility is itself a medical problem regardless of the underlying chronic disease.

## Conclusion

Mobility limitations are highly prevalent with increasing age and are related to negative health outcomes such as hospitalization and falls.

As mobility is a multi-factorial and complex construct, interdisciplinary approaches are mandatory. The lack of a sole definition as well as concepts across disciplines and persons involved are posing barriers for effective mobility prevention. A central aspect in age-related mobility research is the understanding of the interaction of the involved mechanisms, processes, and contributing systems which is complex. Old approaches, e.g., the role of muscle mass, are being questioned and new approaches such as neuromuscular and cognitive processes are coming into focus. Psychological aspects are less investigated, e.g., FrPCs and aging images, as well as behavioral domains, e.g., sedentary behavior with relationships to mobility.

Medical, social, and psychological research is needed for mobility research under the approach of healthy aging.

## Author Contributions

EF designed and wrote the manuscript. RK and CS wrote and reviewed the manuscript. All authors contributed to the article and approved the submitted version.

## Conflict of Interest

The authors declare that the research was conducted in the absence of any commercial or financial relationships that could be construed as a potential conflict of interest.
